# Exploring lay public and dental professional knowledge around HPV transmission via oral sex and oral cancer development

**DOI:** 10.1186/s12889-019-7923-6

**Published:** 2019-11-15

**Authors:** Mario A. Brondani, Adriana B. Siqueira, Claudia Maria Coelho Alves

**Affiliations:** 1 Division of Dental Public Health. Department of Oral Health Sciences, Faculty of Dentistry, University of British Columbia, 2199 Wesbrook Mall, Vancouver, V6T 1Z3 BC Canada; 2 Porto Alegre, Brazil; 3 Department of Dentistry II, Federal University of Maranhão, Av. dos Portugueses, 1966 - Vila Bacanga, CEP- 65080-805 São Luis-Maranhão, Brazil

**Keywords:** Oral sex, HPV, Oncology, Oropharyngeal cancer, Oral cancer, Public forum, Questionnaire, Dentist, Knowledge

## Abstract

**Background:**

Human papillomavirus (HPV) has been associated with certain types of oropharyngeal cancers and yet, the level of knowledge that dental professionals and the lay public have in terms of HPV transmission, oral sexual activities, and oral cancer development needs exploration. The aim of this study was to assess the knowledge held by practicing dental professionals as well as the lay public regarding Human Papillomavirus (HPV) transmission through oral sex and subsequent oropharyngeal cancer development.

**Methods:**

Textual data were collected from a public forum with dental professionals in.

Vancouver, who discussed the HPV-oral sex-oral cancer triad, and from survey data gathered from 212 lay public participants (also in Vancouver) who answered a 13-item questionnaire on the perceived risks of oral sex in terms of HPV infection and oropharyngeal cancer development. The data were analyzed statistically by age group, gender, and sexual orientation using descriptive statistics, while an ANOVA test was used to compare variation in the responses to the survey (*p*-value = 0.05).

**Results:**

The forum engaged 46 health care professionals, many of whom were aware of the potential risks for head and neck cancer development due to HPV infection, while also questioning *“how to effectively talk about HPV with patients.”* The survey revealed that 34.5% of the participants believed that oral sex is an activity with *no or low risk* for the transmission of HPV, while 84% of participants believed the same sexual practices were of *low* or *no-risk* for HIV (Human Immunodeficiency Virus) transmission. Most participants (82%) never discussed oral sexual activities with their physicians or dentists/dental hygienists.

**Conclusions:**

The general public remains mostly unaware of the potential links between HPV infection and oropharyngeal cancer. Physicians and dental providers should discuss oral sexual practice with their patients to raise awareness.

## Background

Oral cancer includes lesions of the lips, tongue, cheeks, gingiva, floor of the mouth, hard and soft palate, sinuses, and pharynx [1]; it has high mortality and morbidity rates if not diagnosed and treated accordingly [2]. Oral cancer has both physical and physiological impacts on quality of life [3, 4]. Approximately 11.2 new cases of oral cancer per 100,000 people are diagnosed every year in the U.S. [5] and about 4600 cases total in Canada [6]. Although the overall incidence of oral cancer is about 12 per 100,000 per year in middle-age men and 5 per 100,000 in middle-age women in Canada [7], reports confirm that the incidence of oral cancer is rising among younger adults [8]. Human Papillomavirus (HPV) seems to play an independent role in the development of almost one-third of all oral carcinomas, particularly oropharyngeal malignancies, to the extent that more than 2000 peer reviewed publications have been produced on the topic over the past decade [9, 10].

The burden of HPV-positive oropharyngeal cancers, compared to other oral cancers, is disproportionately high among men [11]. HPV, as a sexually transmitted pathogen, remains prevalent—to the extent that 3 in every 4 sexually active men and women worldwide are infected by the virus; however, most remain without symptoms [11, 12]. HPV causes virtually all cancers of the cervix in women [13] and anal cancer in men [14]. It is believed that 79 million Americans, most in their late teens and early 20s, are infected with HPV [15]. Most of the existing studies have focused on HPV pathogenesis in cancer development, its treatment, and disease prognosis [2, 7, 10, 11, 16].

From more than 100 different strains of HPV, subtypes 16 and 18 have been associated with the majority of HPV-positive head and neck cancers [7, 17–19]. These two subtypes are also present in most ano-genital and cervical malignancies [11, 15, 20], which might be explained, in part, by changes in sexual habits in recent decades. In fact, the twenty-first century has witnessed a reduction in the age of onset of sexual activity, an increased number of partners and oral sexual practices [21], as well as easier access to online dating sites [22, 23].

With HPV being easily transmitted through skin and mucosal contact, oral sex is seen as a potential vehicle for its transmission from the ano-genital area to the oral cavity [11, 24–27]. However, such potential is not without controversy, given the difficulty to test for such transmission [28].

Nonetheless, individuals with frequent oral sex encounters, a greater number of different sexual partners, and earlier sexual experiences seem to be at a higher risk [29]^1^ for oropharyngeal cancer development [19, 24]; gender and sexual identity also have a heavy influence on sexual practices [30]. Given the high costs of cancer treatment and the often negative psychosocial impact of such treatments when they involve disfigurement, awareness and prevention are key. While prevention remains important, the promising prophylactic vaccination efficacy for oral HPV infection and oropharyngeal cancers is poorly documented since its impact on cancer incidence will not be readily measurable for several decades following immunization [31]. HPV screening also remains limited by a lack of knowledge about the natural history of the disease. Nonetheless, the level of knowledge that dental professionals [32–35] and lay people [35] may hold in terms of HPV transmission through oral sex and its connection to the development of oral cancer have only been explored sporadically. Considering that most individuals infected with HPV had not even heard about this virus [36, 37], we performed a follow-up study from our original studies in 2008 [34] and 2010 [35]. The aim is to further explore the knowledge held by dental professionals, along with the straight and queer (i.e., gay, lesbian, bisexual, questioning, intersex, transgendered referred to herein as *queer*) general public, in regard to HPV transmission through oral sex and the development of oral cancer, using a more comprehensive dataset sample.

## Methods

The University of British Columbia Research Information System (RISe) Ethical Approval was obtained through H07–03121 (for the original study), H16–00735 (for the follow up study reported herein) and H19–01005 (for the hiring of a research assistant and further knowledge dissemination).

### Forum discussion

As described elsewhere [34], a forum was advertised at a local organization that reaches out to dental professionals working in the Vancouver, British Columbia, Canada. The forum occurred in Vancouver at a host community organization; registration was free and limited to 50 participants on a first-come, first-served basis, with dental professionals emailing the research assistant (AS) and expressing their willingness to participate. Preference was given to those who did not participate in the previous forum [34] (upon being asked via email), although there was no mechanism in place to prevent recurrent attendance. The forum was organized so that four experienced clinical researchers with backgrounds in social work, oral medicine and oral pathology, dentistry, and nursing, respectively, highlighted the following topics (Table 1):

- ❖ The incidence, morbidity, and risk factors for oral malignancies;
- ❖ The current understanding around HPV, oral cancer, and oral sexual practices;
- ❖ The roles of health care professionals in educating their patients on the transmission of HPV during the performance of oral sex and its potential association with oral cancer;
- ❖ The individuals at a higher risk, and screening programs available, for oral malignancies;
- ❖ The difficulties in conducting patient education given that sexual practices are usually not discussed in a dental setting.

**Table 1:** ^a^ Summative points regarding oral sex, HPV infection, and oropharyngeal cancer development

General relevance – systemic health	
HPV is a frequently and commonly transmitted infection worldwide	HPV is likely transmitted by skin and mucosal contact *“But how much contact is enough for this transmission to actually occur? I mean, is it a one time enough and you get it or…. is it also about the intensity of contact, say, a touch or else?”* Male dentist
There are more than 120 different strains of HPV (200 strains according to some studies)	More than 40 types of HPV can be isolated in the mouth and genital tracts concomitantly - they are the same strain
It is estimated that about 75% of all sexually active men and women are infected with HPV. The majority will remain asymptomatic, but highly infectious *“Ok, so that means that potentially 3 out of 4 of us here are carrying the HPV virus, right? That is very telling and somewhat scary, really, when you think about it.”* Male dentist	A common clinical manifestation of HPV is a skin wart caused by HPV 1, 2, 3, and 4; Warts are skin growths, not cancers; Genital warts are caused by HPV 6 and 11, different from those causing skin warts; Genital warts are not considered cancers
HPV 16, 18, 31, 35, and 45 do not cause visible warts in genital areas, but have been associated with pre-cancerous lesions in the cervix and anal mucosa	95% of cervical cancers are associated with HPV 16 and 18. In British Columbia, 6 cases of anal cancer in men are diagnosed every year *“It looks like 95% is basically the majority. If transmission is almost inevitable given what it is know, is it actually preventable? Or is something that we will all have at some point, but I understand that also the majority of us will be just fine … reassuring!* Female dentist
HPV prophylactic vaccination appears to be an optimal primary prevention method	The first-generation prophylactic HPV vaccines, Gardasil® and Cervarix® cover the most prevalent high-risk types, HPV 16 and 18; five additional oncogenic HPV types (31, 33, 45, 52 and 58) were added to the existing Gardasil quadrivalent (6, 11, 16, 18) to develop a nanovalent HPV vaccine.
*“We apply anesthetic inside the oral cavity multiple times a day, every day of the week. I read that we could also be offering HPV vaccine, as we do with point-of-care HIV testing. That makes sense, but I do not know how patients would perceive that coming from dental providers.* Male dentist	
Specific relevance – oral health	
Oral sex appears to be a potential vehicle for HPV transmission even though it is difficult to prove	Oral sex is defined as an intimate contact of teeth, gums, lips, and tongue with genital (vagina, scrotum, penis), groin, and anal areas
*“I guess kissing is here too. Well, that is almost the same to say that if you take a breath, you will get it. How can you not? I would be very careful in how people would interpret this. Wasn’t that Hollywood actor that once mentioned that his oral cancer was from oral sex, or something like that? I cannot remember his name now.”* Male dental hygienist	
Some oral cancers (oropharyngeal area) have been associated with HPV infection	HPV 16 and 18 are risk factors for the development of oral cancer, the same strain associated with anogenital malignancies.
As a multifactorial disease, there is no single cause for oral cancer. Risk factors are associated with oral cancer but do not cause it	People infected with HPV are 30 times more likely to develop oral cancer than those who are not *“I had no idea it was that high.”* Female dental hygienist *“Does it matter if you are re-infected? Do those odds increase with reinfection or do they remain the same … once you get it, you are done?* Male dentist
High risk individuals include those with more than 5 different oral sexual partners during a life-time and earlier oral sex experience	High frequency of oral sex, about 3 to 5 times a week in the past 30 days, increases the risk for developing oral cancer 9-fold
Patients might not feel comfortable discussing oral sex practices with a dentist	Patients might feel that oral sex practices are not meant to be discussed in a dental office
*“That is tricky. I mean, can we comfortably talk about that [oral sex] during a dental appointment? I certainly do not feel that way, and with a male patient even less so… perhaps not at the first visit, like, to ‘spill the beans’ like that. Also, what about the one timers, say, those patients that come for a consult or emergency treatment and we never see them again? Where does the responsibility lie?* Female dentist	

^a^Adapted from Brondani, Cruz-Cabrera and Colombe, 2010. The facts listed in this table might or might not reflect the current knowledge on the topic as it combines both experts’ opinion and lay understandings. It does not necessarily concur with the published literature.

During the forum, the authors (MB and AS) took textual field notes on the participants’ comments so that a more accurate representation of their accounts could be portrayed in Table 1, with italics. These verbatim notes, taken as accurately as possible, were used to illustrate ideas shared by the participants when discussing the issues at hand with the presenters. However, participants were not identified by name or age, just gender and professional role. The textual information was not analyzed thematically for codes and themes, as is normal for a traditional qualitative research inquiry. Since the forum presented in this study focused on similar topics as the previous one [34], overlap of information might have occurred.

### Lay public survey

Potential participants were informed about the survey from posters placed at three large community health centres in Vancouver, as described elsewhere [35]; participants had to be older than 19 years of age, be of any gender or sexual orientation, and able to understand English sufficiently to answer the survey questions. One hundred and fifty copies of the questionnaire were distributed to each of the centres (total of 450 surveys), which also collected the surveys. The total number of potential participants reached at each community health centre is unknown; a minimum sample of 200 was estimated for the survey to yield significant results. A brief 13-item multiple-choice anonymous questionnaire was developed from a previous study by the leading author [35], and covered issues around:

- ■ Frequency of oral sex activities for the past month: daily, up to 3 times a week, once a week, a few times a month, once a month, rarely;
- ■ Risk of transmission of HPV, HIV, and other STIs through oral sex: no risk, low risk, moderate risk, high risk, don’t know;
- ■ Risk of developing oral cancer through oral sex: no risk, low risk, moderate risk, high risk, don’t know;
- ■ Number of different oral sex partners for the past month: my partner only, up to five different persons, more than five different persons;
- ■ Use of protective measures while engaging in oral sexual practices: yes, no;
- ■ Visit to a dentist and/or a physician over the past year: yes, no;
- ■ Have been asked about oral sexual practices by a dentist and/or a physician over the past year: yes, no;

The question about oral cancer development was framed “as is”, without distinguishing the various types of oral and pharyngeal malignancies; it was used as an encompassing term for all oral and pharyngeal cancers. After a 3-month period, all hardcopies of the questionnaires (completed or not) were collected by the research assistant (AS). Data were analyzed statistically for comparisons between three age groups (19–30, 31–50, > 50), gender (male, female), and sexual orientation (queer, straight). As an exploratory study, data were analyzed with descriptive statistics, while an ANOVA test was used to compare variation in the responses to the survey (*p*-value = 0.05).

## Results

This manuscript focused on the information gathered from a follow-up study, while the discussion included the previous data on the same topics when appropriate [34, 35]. The forum fostered open dialogue and involved 46 participants in total: dentists (*n* = 19), dental hygienists (*n* = 21), and certified dental assistants (*n* = 6). As mentioned by a female dental hygienist, *“this topic is important and should be readily and openly discussed among ourselves and also with the public given the impact and implication of an oral cancer diagnosis – the issue that still remains for me is ‘how to effectively talk about it’ with our patients.”* The other main points discussed during this forum, from the perspectives of the presenters and the dental professionals, are outlined in Table 1, together with some verbatim comments made by the participants when discussing each of the respective topic; some comments included HPV vaccination as an important primary prevention strategy and the barriers in bringing up the issue of oral sex and HPV transmission during a dental appointment.

Two hundred and twelve participants completed the questionnaires gathered from the three community centres; the number of returned questionnaires were roughly distributed evenly among the centres (47% completion rate from the 450 questionnaires distributed). Forty-nine respondents self-identified as *queer* (an umbrella term for gay, lesbian, bisexual, questioning, or transgendered); of these 49 respondents, 57% were between the ages of 19 and 30, while 15% were older than 50. Among the participants older than 50 years of age, 65% of queer self-identified men and 73% of straight self-identified men reported oral sexual practices exclusively with their current partners, whereas 74% of queer self-identified women and 88% of straight self-identified women from all ages reported identical behaviour (data not shown). Overall, younger men and women between 19 and 30 years of age, of any sexual orientation, reported having had more than one oral sex partner over the last month, compared to older participants.

Eighty-five percent of all participants (180 respondents) believed oral sexual practices to be of *low* or *no risk* for HIV transmission, while 73 respondents (34.5%)—57.8% of these being younger than 50 years of age— regarded oral sexual practices as being of *no* or *low risk* for the transmission of HPV (Table 2). More than 80% of respondents perceived the same activity to be of *moderate* to *high risk* for other sexually transmitted infections (responses not mutually exclusive). Nobody perceived oral sexual practices to be of *no risk* for the transmission of sexually transmitted infections. For the risk of oral cancer development, more than 70% of all respondents (*n* = 150, mostly younger than 30 years old) believed that oral sexual practices posed *no risk*, while less than 30% of the respondents *did not know* about such risks. The answers for these questions in the three age groups did not vary significantly (ANOVA, *p* = 0.85). Ninety-one percent of respondents (*n* = 193) reported not utilizing any form of protection while engaging in oral sex activities.

**Table 2:** ^d^ Age range of participants distributed according to perceived risk for HIV, STIs, HPV and oral cancer development (in general) regarding oral sex

Question	Age group and number of participants^a^			
	19–30 *(57%)*	31–50 *(28%)*	> 51 Total *(15%)*	*P* values^b^
	121	59	32,212	
For the transmission of HIV,do you think oral sex is an activity of**:**				
No/low risk	113 (93.3%)	48 (81.3%)	19,180 (59.3%) (84.9%)	<.09
Moderate/ high risk perceived	8 (06.6%)	11 (18.6%)	13 32 (40.6%) (15.1%)	
For the transmission of sexually transmitted infections^c^, do you think oral sex is an activity of:				
Low risk	11 (9.1%)	15 (25.4%)	11 37 (34.4%) (17.5%)	
Moderate/ high risk	110 (90.9%)	44 (74.5%)	21,175 (65.6%) (82.5%)	
For the transmission of human papillomavirus (HPV), do you think oral sex is an activity of:				
No/low risk	33 (27.3%)	18 (30.5%)	22 73 (68.7%) (34.5%)	<.05
Moderate/ high risk	88 (72.7%)	41 (69.5%)	10,139 (31.3%) (65.5%)	
For the development of oral cancer, do you think oral sex is a risk?				
No	79 (65.2%)	47 (79.6%)	24,150 (75.0%) (70.7%)	<.01
Yes	1 (00.8%)	1 (01.7%)	0 2 (0.1%)	
Don’t know	41 (34.0%)	11 (18.7%)	8 60 (25.0%) (28.3%)	

^**a**^ Percentages in brackets refer to the total for that age group

^**b**^ *P*-values shown when applicable

^**c**^ Sexually Transmitted Infections include infections such as chlamydia, gonorrhoea and syphilis

^d^Adapted from Brondani, Cruz-Cabrera and Colombe, 2010.

From all participants, 80.5% (171 respondents) reported having had a medical and a.

dental appointment at least once in the previous year. During these visits, 82% of the respondents were never asked about oral sex practices by their physicians. This percentage reached 94.7% (161 participants out of 171 who had a dental appointment in the previous year) for those whose dentists did not ask about the same practices in that appointment.

## Discussion

As most scientific studies on head and neck cancers associated with HPV emphasize pathophysiology and treatment [9, 10, 16, 24, 38, 39], this research focused on the level of awareness dental professionals and the lay public hold about the interplay among HPV, oral sex, and oral cancer, particularly in the oropharynx, following on from two of our studies conducted a decade ago [34, 35]. It is important to understand such awareness, so that the gap between scientific information and everyday knowledge is decreased [36, 37]. As discussed previously, the forum highlighted that oral cancer prevention in general must move away from focusing solely *“on tobacco, age, alcohol consumption, dietary habits, and oral hygiene practices”* to also ask about *“oral sex practices and all of the potential pathogens associated with it”* [34]. The potential effect of HPV infection on oropharyngeal cancer development via oral sexual practices became well publicized when an American actor made headlines in 2013 by initially saying that his throat cancer was caused by oral sex, to later retract his comments [40]. And it has been only recently that oral sex was brought “officially” into the discussion [26], despite previous efforts [29, 32–37]. Perhaps not surprisingly, many participants from the follow-up survey, like their counterparts [34, 35], did not believe in the possibility of transmission in this manner. There is a continued need to include HPV infection in mainstream discussions, given that less than one-third of sexually active individuals infected by this virus have actually heard about it [29, 41, 42].

The American actor’s headline and public websites can be used as tools to facilitate the discussion of this topic with patients. In fact, the need for such a discussion was brought up by a participant at the forum who voiced that “*we should consider how to better start the conversation around HPV with a patient without feeling awkward about sexual practices”.* The time has come to openly converse with patients about other potential risks factors for oral cancer development, aside from tobacco (chewed or smoked) and excessive alcohol consumption; however, not everybody feels ready or comfortable to initiate such a conversation, as alluded by the above participant and found by studies around other sensitive subjects like HIV point-of-care [43] and substance use [44]. Nonetheless, this suggestion is justified by the fact that most physicians (82%) and dentists (94.7%) never talked about potential HPV infection via oral sexual practices with the patients from this follow-up study, nor did their counterparts more than a decade ago [35]. Dentists may have recently acquired some knowledge about the potential associations between HPV and oropharyngeal cancers, but they still lack the skills to have a conversation to educate patients effectively and without embarrassment [45]. As advised by Martin-Hernan and colleagues [46], “*dentists, as health professionals, ought to get more heavily involved in the detection of these factors … especially in young patients with no risky habits traditionally linked to oral cancer, such as tobacco and/or alcohol consumption.”*

The surveyed participants agreed with our previous study [35] and considered oral sex to be a *low risk* activity for oral cancer (general term used) and of *moderate* to *high risk* for other sexually transmitted infections, regardless of their sexual orientation or age. Participants in this and the previous study [35] might have considered the act of engaging in oral sex itself as a potential risk, and therefore, judged it to be of low or no risk. If respondents felt that oral sex involves low or no risk for oral cancer (general term used) regarding HPV transmission, it is no surprise that the vast majority (91%) of the participants did not make use of any protective measure during oral sex activities (results not shown); similar finding were observed in another study [47]. Furthermore, the use of protection as a preventive measure for transmission of certain pathogens during the practice of oral sex has been questioned given the fact that HPV does spread via skin-to-skin and mucosal contact and even beyond the genital or anal area [24]. Therefore, because of that, “*potentially 3 out of 4 of us here are carrying the HPV”,* as voiced by one of the forum participants (Table 1). Prophylactic HPV vaccination, on the other hand, surfaces as promising protection against the development of oropharyngeal cancers [30, 31], as some vaccines cover nine subtypes of HPV, including 16 and 18 [48]. In fact, recent studies have advocated for offering the HPV vaccine in dental settings, which might help increase the currently low levels of coverage and uptake [49] but there is need to explore the extent to which oral health providers would be willing to offer such vaccine in Canada.

Nonetheless, the gap we highlighted above does indeed seem to exist between peer-reviewed information (very few individuals outside a research or clinical setting have access to, or are interested in reading, scientific publications) and lay public awareness. Knowledge transfer continues to fall short in advising the community at large about studies linking HPV with some oral cancers. Hence, as found in this study and elsewhere [34, 35], health care providers, particularly dentists, are not openly discussing this issue with their patients [32, 50] (probably because of HPV knowledge deficits [31, 47]) even though dental professionals seem to agree about the importance of discussing HPV with their patients [51, 52]. As found by others [34, 35, 45, 46, 52], this study reinforces the need for educational awareness for the public. Once provided with consistent information, they should be able to take control of their sexual practices and health. Awareness and educational messages should also target gender and sexual orientation in a way as to be inclusive, yet respecting inherent differences among these groups.

There are a number of limitations in this study. One of which is the single forum discussion consisting of dental professionals only, even though it builds on previous events [35]. Also, the convenient sampling strategy used to recruit lay public respondents for this follow-up study, predominantly younger participants from one urban city, compromises generalizability. A larger sample size is warranted to include adults and older adults, for example, and participants from various cultural and socioeconomic backgrounds. Furthermore, a more qualitative study should follow in order to unravel the reasons behind the knowledge and beliefs expressed by the participants in this study; this would allow an informed dialogue to take place between health care providers and their patients.

## Conclusions

Discussions around HPV and head and neck cancers have mostly focused on the pathophysiology and treatment of such malignancies. Given that the transmission of HPV to the mouth likely occurs via sexual activities, there is an increased need to foster a dialogue between health care providers, especially dentists, and their patients about risk factors while leaving aside the embarrassment. This dialogue is still not happening, and participants remain unaware of the potential associations between HPV infection and oral cancer. The follow-up forum and survey results presented herein can be replicated by other researchers and disseminated to a larger group of participants, contributing to capacity building and training of health care providers so that a dental chair-side conversation about risk factors for head and neck cancers can be fully optimized.

## Data Availability

The datasets generated and analyzed for the current study are not available publicly due to participant confidentiality, but may be obtained from the corresponding author upon reasonable request.

## Abbreviations

ANOVA: Analysis of variance
HIV: Human immunodeficiency virus
HPV: Human Papillomavirus
RISE: Researcher information services
STIs: Sexually transmitted infections

## Footnotes

1 High-risk participants were defined as those having 4 or more different oral sex partners for the last 30 days, whereas low risk included those with up to 3 different partners for the same period. High frequency of oral sex (3 to 5 times a week in the past 30 days) increases the risk for developing oral cancer 9-fold [28].
